# Winkelstabile Plattenosteosynthese bei Patellafrakturen: Technik und Umsetzung in der Praxis

**DOI:** 10.1007/s00113-025-01646-y

**Published:** 2025-10-29

**Authors:** Julian Kylies, Jannik Frings, Karl-Heinz Frosch, Matthias Krause

**Affiliations:** 1 Department of Trauma and Orthopaedic Surgery, University Medical Center Hamburg-Eppendorf, Martinistraße 52, 20246 Hamburg, Deutschland; 2 Department of Trauma Surgery, Orthopaedics and Sports Traumatology, BG Klinikum Hamburg, Hamburg, Deutschland

## Einleitung

Die operative Versorgung von multifragmentären, dislozierten Patellafrakturen kann insbesondere bei komplexer Frakturmorphologie oder bei osteoporotischer Knochenstruktur eine chirurgische Herausforderung darstellen. Winkelstabile Plattensysteme bieten hier eine biomechanisch überlegene Alternative zur klassischen Zuggurtungs- oder isolierten/kombinierten Schraubenosteosynthese. Ziel dieses Beitrags ist die praxisnahe Darstellung der Operationstechnik, inklusive präoperativer Planung, Durchführung und Nachbehandlung.

## Indikationen

Die Indikation zur Plattenosteosynthese ergibt sich bei dislozierten, mehrfragmentären Frakturen mit Stufenbildung ≥ 2 mm insbesondere bei eingeschränkter Knochenqualität. Auch Frakturen mit multifragmentärem distalem Polausriss, welche durch konventionelle Techniken unzureichend adressiert werden können, profitieren von winkelstabilen Plattensystemen.

## Kontraindikationen

Eine absolute Kontraindikation besteht bei kontaminierten oder infizierten Weichteilen sowie bei unzureichender Weichteildeckung über der Patella. Relative Kontraindikationen umfassen systemische Infektionszeichen, mangelhafte Compliance oder schwere Begleiterkrankungen mit erhöhtem Operationsrisiko.

## Vorteile der Plattenosteosynthese

- Hohe Primärstabilität, auch bei eingeschränkter Knochenqualität
- Biomechanische Überlegenheit gegenüber der Zuggurtungs- und reinen Schraubenosteosynthese, insbesondere bei multifragmentärer Frakturmorphologie
- Möglichkeit zur sicheren Fixation, auch von distalen Polfragmenten
- Niedrige Revisionsrate und gute funktionelle Langzeitergebnisse

## Nachteile der Plattenosteosynthese

- Höherer Implantatpreis im Vergleich zu klassischen Verfahren
- Fehlende Kompression über die Platte bei einfachen Frakturen
- Technisch anspruchsvoller Eingriff mit notwendiger Expertise
- Gegebenenfalls Risiko postoperativer Weichteilirritationen durch das Implantat

## Präoperative Planung und Aufklärung

**Abb. 1:**
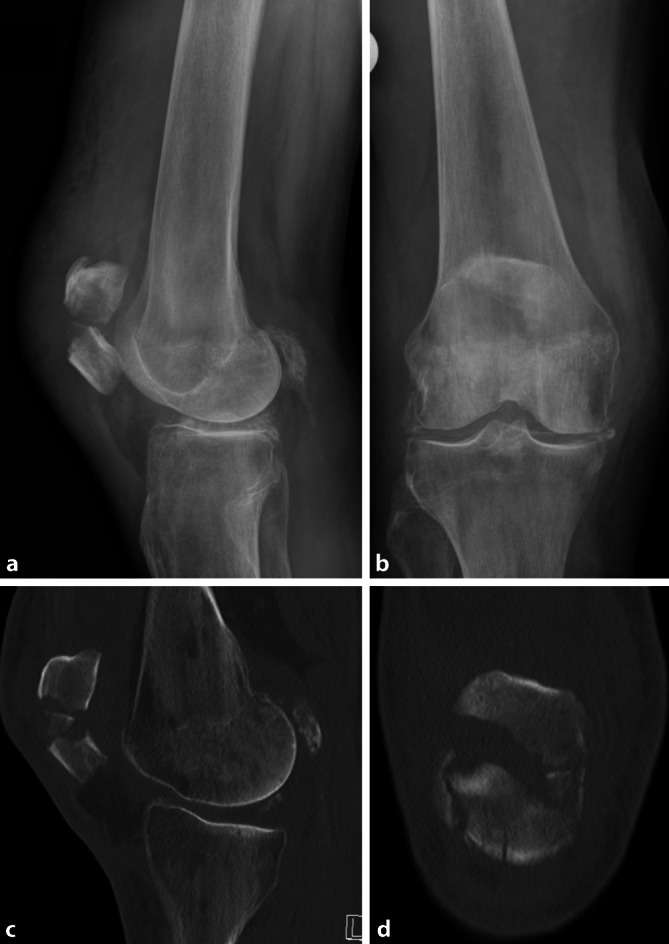
Die vorliegende Bildgebung zeigt den Befund einer 70-jährigen Patientin, die in der Häuslichkeit auf das rechte Knie gestürzt ist. Die präoperative Basisdiagnostik bei Patellafrakturen besteht aus einer initialen Röntgenbildgebung (**a,** **b**), gefolgt von einer computertomographischen Evaluation (**c,** **d**). In diesem Fall zeigt sich eine mehrfragmentäre dislozierte Patellafraktur mit Beteiligung des distalen Pols (AO-Typ 34-C3). (© Mit freundlicher Genehmigung von PD Dr. Jannik Frings.)

Zur präoperativen Planung gehören ein konventionelles Röntgen in 2 Ebenen (Abb. 1a, b) sowie eine CT-Untersuchung zur differenzierten Beurteilung der Frakturmorphologie (Abb. 1c, d). Bei kompliziertem multifragmentärem Frakturmuster kann ein präoperatives MRT zur Detektion begleitender Band- und Knorpelverletzungen sinnvoll sein.

Im Aufklärungsgespräch sollten neben allgemeinen Risiken folgende spezifische Aspekte thematisiert werden:

- Risiko einer sekundären Dislokation oder unzureichenden Reposition,
- Notwendigkeit eines Revisionseingriffs bei verbliebener Gelenkstufe > 2 mm,
- Möglichkeit postoperativer Bewegungseinschränkung oder Arthrofibrose,
- implantatbedingte Irritationen und Notwendigkeit einer Materialentfernung im Verlauf.

## Operationsablauf

### Lagerung und Anästhesie

Die Operation erfolgt in Rückenlagerung in Spinal- oder Allgemeinanästhesie. Das betroffene Bein kann in einem elektrisch einstellbaren Beinhalter oder auf einer sterilen Rolle positioniert werden. Eine Blutsperre (250–350 mm Hg) kann fakultativ eingesetzt werden, ist aber mit einem erhöhten Nachblutungs- und Infektionsrisiko assoziiert. Die intraoperative Bildwandlerkontrolle (a.-p., seitlich, tangential) ist essenziell zur Beurteilung der Reposition und Implantatlage.

### Zugang

**Abb. 2:**
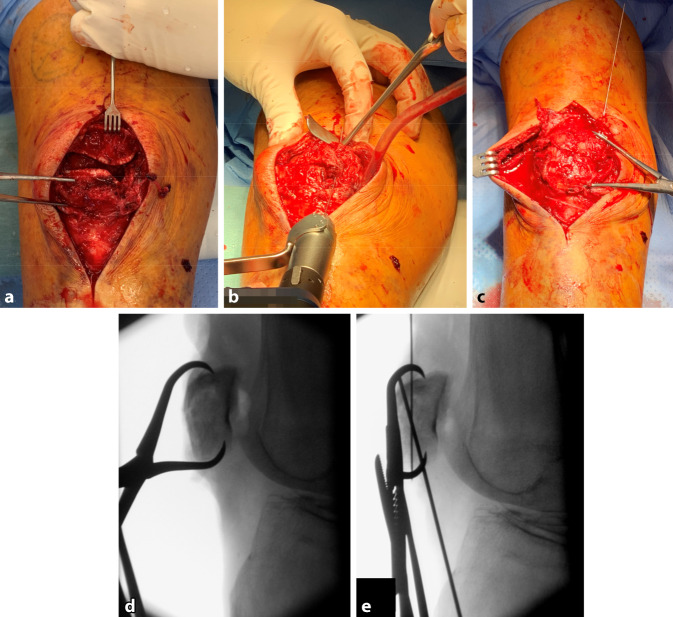
**a** Die Präparation erfolgt über einen zentralen Längsschnitt und die Fraktur kommt zur Darstellung. **b,** **c** Die Reposition erfolgt mittels scharfer Repositionszange und temporärer Kirschner-Draht-Fixierung. **d,** **e** Die anatomische Reposition wird fluoroskopisch kontrolliert. Es zeigt sich eine anatomische Reposition. (© Mit freundlicher Genehmigung von PD Dr. Jannik Frings.)

Der Zugang erfolgt über einen zentralen Längsschnitt über der Patella. Nach Inzision und bei Zerreißung ggf. Resektion der Bursa praepatellaris wird der Streckapparat dargestellt (Abb. 2a). Besonderes Augenmerk gilt dem R. infrapatellaris nervi sapheni, um postoperative Hypästhesien und die Bildung eines Neurinoms zu vermeiden. Die oberflächliche Faszie über der Patella sollte erhalten bleiben, um sie zum Schutz über der Platte verschließen zu können. Im Falle einer multifragmentären Frakturmorphologie empfehlen wir als primäres Ziel die nahezu anatomische Gelenkflächenrekonstruktion über eine direkte Gelenkflächendarstellung mittels Arthrotomie und Patella-Eversion. Aufgrund der v. a. mediodistal einlaufenden Durchblutung empfehlen wir die laterale Arthrotomie mit oder ohne Z‑Plastik, um das Risiko einer sekundären Fragmentnekrose zu reduzieren. Insbesondere bei medialer Trümmerzone sollte die Arthrotomie bis an die Tuberositas und proximal entlang der Quadrizepssehne unterhalb des M. vastus lateralis erfolgen. Die Patella lässt sich dadurch in der Regel gut evertieren, sodass ein direkter Blick auf die gesamte Gelenkfläche mit optimaler Repositionskontrolle erfolgen kann.

Sollte im späteren Verlauf die Implantation einer Endoprothese erforderlich sein, kann diese über den gleichen Zugang implantiert werden.

### Frakturpräsentation und Reposition

Nach Entfernung des Frakturhämatoms und Débridement erfolgt die anatomische Reposition der Frakturfragmente. Im Fall der Patella-Eversion erfolgt die Gelenkflächenrekonstruktion von artikularseitig unter Schonung des Hoffa-Fettkörpers und der Patellasehne. Die Reposition wird mithilfe von spitzen Repositionszangen und temporären Kirschner-Drähten stabilisiert (Abb. 2b, c). Während der Reposition erfolgt die fluoroskopische Qualitätskontrolle der artikulären Rekonstruktion mit genauer Überprüfung von Gelenkstufen im Bereich aller 3 Facetten (medial, lateral, First) (Abb. 2d, e).

### Plattenanpassung und Fixation

**Abb. 3:**
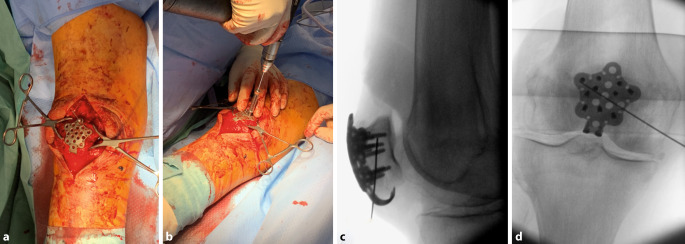
**a** Nach der Reposition wird eine Platte in geeigneter Größe auf der Patella positioniert und temporär mittels Olivendraht fixiert. **b** Es erfolgt sodann das Einbringen winkelstabiler monokortikaler Schrauben, idealerweise mindestens 3 Schrauben proximal und 3 Schrauben distal der Fraktur. **c,** **d** Es erfolgt die fluoroskopische Kontrolle der Schraubenlänge in 2 Ebenen. (© Mit freundlicher Genehmigung von PD Dr. Jannik Frings.)

Es existiert eine Bandbreite unterschiedlicher Plattenkonzepte und -designs zur Versorgung von Patellafrakturen. Den meisten gemeinsam ist die zentrale Positionierung auf der Vorderfläche der Patella (Abb. 3a, b). Die Auswahl der Plattengröße richtet sich vordergründig nach der Adressierungsmöglichkeit relevanter Fragmente, über die zur Verfügung stehende Plattenlochkonfiguration. Ein geringer Plattenüberstand um wenige Millimeter proximal oder distal ist hier zu vernachlässigen, lateral oder medial kommt es hingegen schnell zu Weichteilirritationen.

**Abb. 4:**
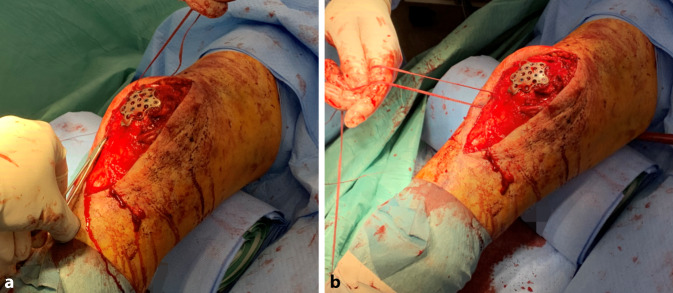
Moderne Plattensysteme bieten die Möglichkeit, kleinere Fragmente beispielsweise mittels McLaughlin-Schlinge zu fixieren. Begleitverletzungen der Quadrizeps- oder Patellarsehne können durch Augmentationsnähte ebenfalls direkt an der Platte gesichert werden (**a,** **b**). (© Mit freundlicher Genehmigung von PD Dr. Jannik Frings.)

Die temporäre Fixierung erfolgt mittels monokortikaler Kirschner-Drähte oder Olivendrähte. Nach fluoroskopischer Kontrolle wird die Platte über winkelstabile Schrauben fixiert (Abb. 3c, d). In der abschließenden fluoroskopischen Dokumentation ist neben der Reposition und der Plattenlage die adäquate Schraubenlänge zu beachten, um eine intraartikuläre Lage zu vermeiden. Optional kann eine zusätzliche McLaughlin-Schlinge zur Fragmentfixation eingebracht werden. Begleitverletzungen der Quadrizeps- oder Patellarsehne können durch Augmentationsnähte direkt an der Platte gesichert werden (Abb. 4).

### Wundverschluss

**Abb. 5:**
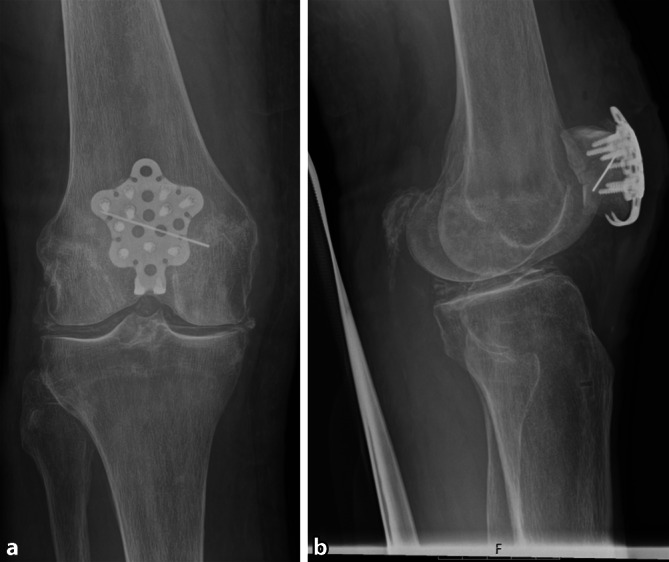
Das postoperative Röntgenbild dokumentiert eine regelrechte Plattenlage, Schraubenlänge und Frakturreposition (**a,** **b**). (© Mit freundlicher Genehmigung von PD Dr. Jannik Frings.)

Nach finaler Bildwandlerkontrolle erfolgt der Verschluss der lateralen Retinacula und der Faszie über der Platte. Eine Subkutannaht und der Hautverschluss folgen konventionell. Abschließend erfolgt die Anlage eines sterilen Verbandes und elastischer Wickelung. Postoperativ erfolgt die erneute röntgen-, ggf. CT-morphologische Kontrolle (Abb. 5).

## Nachbehandlung

Eine vorsichtige Mobilisierung des Kniegelenks erfolgt über 6 Wochen mithilfe einer Bewegungsorthese bei einer Teilbelastung von 20 kg. Eine schrittweise Steigerung der Flexion um jeweils 30° alle 2 Wochen wird empfohlen. Allerdings ist die Belastungsstabilität v. a. bei älteren Patientinnen und Patienten mit fraglicher Compliance bezüglich der empfohlenen Teilbelastung oder Bewegungslimitierung bereits bei der osteosynthetischen Versorgung zu berücksichtigen. Insbesondere in diesem Patientenkollektiv kann auch die vorsichtige schmerzadaptierte Vollbelastung in einer angelegten Streckschiene erfolgen.

## Fehler, Gefahren und Komplikationen

Trotz der hohen biomechanischen Stabilität und der guten klinischen Ergebnisse ist die winkelstabile Plattenosteosynthese nicht frei von Komplikationspotenzial. Zu den häufigsten Fehlern zählt eine unzureichende Reposition mit verbliebener Gelenkstufe ≥ 2 mm. Diese kann zu einer postoperativen Arthrose und persistierenden Beschwerden führen und sollte intraoperativ ausgeschlossen werden. Auch eine unzureichende Schraubenplatzierung, insbesondere im Bereich des Frakturspalts oder mit zu geringer Fixation des proximalen oder distalen Pols, kann zu einer sekundären Dislokation führen. Ein weiteres Risiko liegt im zu frühen postoperativen Mobilisierungsbeginn ohne ausreichende Bewegungsführung oder Schutz vor Beugebelastung.

Postoperative Komplikationen umfassen Hämatombildung, Infektionen, Wundheilungsstörungen sowie Weichteilirritationen durch das Implantat im Langzeitverlauf. In seltenen Fällen kann es zur Entwicklung einer Arthrofibrose kommen. Die Implantatentfernung ist in der Regel nicht erforderlich, sollte aber bei lokalen Beschwerden oder mechanischen Irritationen erwogen werden.

## Fazit

Die winkelstabile Plattenosteosynthese scheint eine vorteilhafte Methode zur Versorgung komplexer Patellafrakturen zu sein. Sie erlaubt meist eine frühfunktionelle Nachbehandlung mit geringen Komplikationsraten. Eine präzise Reposition und systematische Schraubenplatzierung sind entscheidend für das funktionelle Outcome.

